# UHPLC: The Greening Face of Liquid Chromatography

**DOI:** 10.1007/s10337-013-2434-6

**Published:** 2013-03-07

**Authors:** Judyta Cielecka-Piontek, Przemysław Zalewski, Anna Jelińska, Piotr Garbacki

**Affiliations:** Department of Pharmaceutical Chemistry, Faculty of Pharmacy, Poznan University of Medical Sciences, Grunwaldzka 6, 60-780 Poznan, Poland

**Keywords:** Column liquid chromatography, UHPLC, Pharmaceutical analysis, Green approach

## Abstract

Pharmaceutical analysis based on chromatographic separation is an important part of studies aimed at developing routine quality analysis of drugs. High-performance liquid chromatography (HPLC) is one of the main analytical techniques recommended for drug analysis. Although it meets many criteria vital for analysis, it is time-consuming and uses a relatively high amount of organic solvents compared to other analytical techniques. Recently, Ultra-high-performance liquid chromatography (UHPLC) has been frequently proposed as an alternative to HPLC, which means introducing an environment-friendly approach to drug analysis achieved by reducing the consumption of solvents. It also offers greater chromatographic resolution and higher sensitivity as well as requiring less time due to faster analysis. This review focuses on the basics of UHPLC, compares that technique with HPLC and discusses the possibilities of applying UHPLC for the analysis of different pharmaceuticals and biopharmaceuticals.

## Introduction

In view of the obvious need to protect the natural environment and the introduction of ever stricter quality requirements regarding analytical procedures used in pharmaceutical analysis, it is by all means justified to search for analytical techniques in order to meet those requirements.

At present, most to analytical methods recommended by pharmacopoeias are based on chromatographic techniques, of which HPLC is the most common [1–3]. Although the content of organic phase is limited owing to the reversed phase, the wide spread use of this method highlights the necessity to seek less harmful solutions [4, 5].

One of the principles of the environment-friendly approach to solutions used in analytical chemistry is to ensure the universality and availability of instrumental analytical techniques. As far as pharmaceutical analysis is concerned, the rule Reduce-Replace-Recycle (3Rs) appears to be the most relevant [6–9]. In chromatographic analysis for pharmaceutical industry, the principle of replacing and recycling is the one towards which the majority of researchers are oriented at the moment. That is achieved in the development of analytical methods by replacing toxic solvents with those of lower toxicity and by recycling organic solvents. However, the reduction of organic fraction in chromatographic analysis is difficult during the separation of optical isomers.

The achievement of the 3Rs rule may be possible by using advanced analytical techniques such as ultra-high performance liquid chromatography (UHPLC) and supercritical fluid chromatography (SFC) [10–15].

Taking into consideration the necessity of proving the equivalence of newly proposed methods and the need to ensure the transfer of analyte determination, it has been demonstrated that UHPLC is close to conventional chromatography with respect to the operating principles.

The first commercially available UHPLC system was introduced in 2004. Over ten last years the frequency of using UHPLC for analyzing pharmaceuticals and biopharmaceuticals has increased significantly, which indicates that UHPLC and HPLC are transferrable.

The shorter run-time of UHPLC methods results in reducing organic solvent volumes and the whole time of analysis, without decreasing the sensitivity and resolution of determination. Moreover, laboratory staff are less exposed to toxic agents and solvolysis of analytes caused by the presence of organic solvents is limited. Taking into consideration the above mentioned advantages UHPLC methods may be recommended for studies adopting a green approaches to pharmaceutical analysis.

## The Clue to UHPLC

**Fig. 1:**
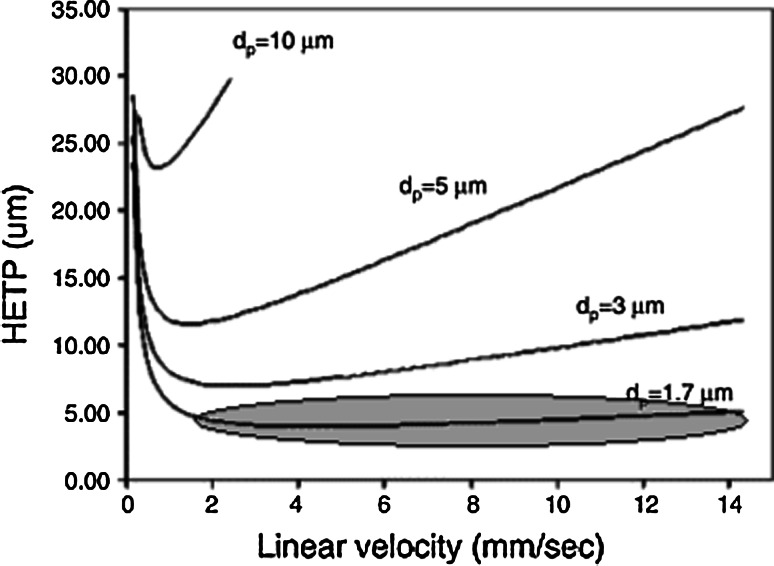
Van Deemter curves for different particle sizes

The efficiently of determination in chromatographic procedures is influenced by the number of theoretical plates. The relationship between the height equivalent to a theoretical plate (HETP) and linear velocity is described by the van Van Deemter equation:

$$ H = Ad_{p} + \frac{{BD_{M} }}{\mu } + \frac{{Cd_{p}^{2} \mu }}{{D_{M} }}, $$

where HETP is denoted as *H*, *A*—Eddy-diffusion coefficient, *d*_*p*_—the particle size of the stationary phase, *B*—longitudinal diffusion coefficient, *μ*—the linear velocity of the mobile phase, *D*_*M*_—analyte diffusion coefficient and *C*—resistance to mass transfer coefficient. The relationship HETP = f(μ) is a hyperbolic function for a stationary phase of particle size greater than 3 μm. When the particle size of column is less than 2 μm, the quotient $\frac{{{\text{d}}H}}{{{\text{d}}\mu }}$ for the distance of HETP = f(μ) is 0. Hence, the use of high linear velocities does not influence the values of HETP (Fig. 1).

The application of stationary phases with smaller packages solves the problem of changes in plate heights. As the mobile phase overcomes the resistance of the stationery phase, UHPLC and HPLC require different pressures. They are proportional to the square of the column package size. Therefore, different ranges of pressure are used in HPLC and UHPLC systems. During the mobile phase flow under a high pressure heat energy is released, which impacts chromatographic separation. High pressure values due to smaller particle sizes also lead to frictional heating. L. Nováková et al. evaluated frictional heating in a UHPLC method under gradient elution conditions during analysis of basic, acidic and neutral drugs using 1.0 and 2.1 mm columns in the range 100–1,000 bar. It was suggested that by decreasing the external temperature a UHPLC method it was possible to achieve the required pressure ensuring the desired column efficiency. As an alternative solution during the method transfer from HPLC to UHPLC, a slight adjustment of the gradient slope was proposed [16]. The resulting significant temperature gradients particularly reduce the efficiency of short columns. As a result of smaller mobile phase volumes and faster mobile phase flows, short injection cycle times and low injection volumes are necessary. The formation of axial (longitudinal) and radial temperature gradients may be the reason for a significant loss of separation efficiency. The temperature distribution across the column also depends on controlling the external temperature of the column wall. The sensitivity of UHPLC is 2–3 times higher (depending on the detection technique) than that observed during HPLC separation [17–20]. It is necessary to take into account the fact that during the analysis of thermolabile drugs the results may not be reliable due to local frictional heating. It is possible that UPC2 will be a better solution for the analysis of non-volatile and thermally labile compounds, as it offers lower analysis temperatures and a significant improvement in run time [21].

**Fig. 2:**
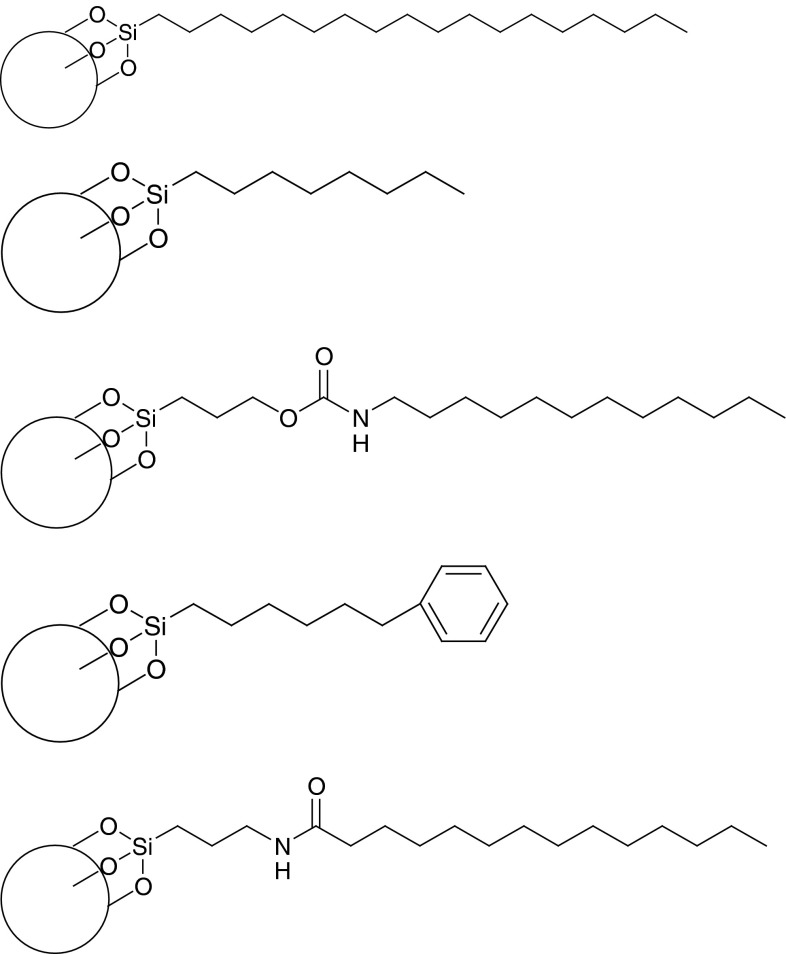
The examples of column filling used in UPLC

The exposure of the stationary phase to high pressure has led to the development of columns with greater resistance to the effect of high pressure. Currently columns which can be used for UHPLC analysis are not offered by many manufacturers. Nonporous columns packed with 1.5 μm particles have the disadvantages of poor loading capacity. Silica-based particles are sensitive to pH changes in the mobile phase and, consequently, basic analytes may cause peak tailing. Columns packed with 1.8 μm particles, dedicated for low-pH operation are also available, for example Zorbax StableBond C8 and C18 columns. It was proved that these columns demonstrate a desired efficiency resulting from increasing the particle size [1]. Another approach when dealing with low pH mobile phases is to use platinum columns with 1.5 μm particles—C8, C18 and extended polar selectivity phases available as silica-based columns with a 100 Å pore size. Platinum columns provide adequate separation, especially in the case of LC–MS analysis, where mobile phases consist mostly of organic solvents. The pH limitations of the mobile phase may also be remedied by the application of polymeric columns. Unfortunately those columns demonstrate low efficiency, limited loading capacity and poor mechanical strength. The introduction of hybrid columns (XTerra^®^-2000, Waters) combines the advantages of silica and polymeric fillings, encouraging the development of a bridged ethyl hybrid (BEH) technology mainly based on the application of fillings containing bridged ethylsiloxane/silica hydrid (BEH): C18, C8, shield C18, phenyl and amide stationary phases (Fig. 2) [22–25]. The majority of those columns are also more resistant to changes in the mobile phase pH.While transferring methods from HPLC to UHPLC the column length and the particle size (*L/dp*) as well as the flow-rate should be considered. Under isomeric elution, the relationship between the flow rates of the mobile phase, the injected volume and the total analysis time are described as follows:

$$ tR_{2} = tR_{1} \cdot \frac{{F_{1} }}{{F_{2} }} \cdot \frac{{V0_{2} }}{{V0_{1} }} $$

where $t_{{R_{2} }}$ and $t_{{{\text{R}}_{1} }}$ are the total analysis times of the methods involved, *F*_1_ and *F*_2_ are the flow rates of the mobile phases and $V_{{0_{2} }}$, $V_{{0_{1} }}$ are dwell volumes established for those methods, respectively [26]. That relationship should be introduced as an adjustment into the gradient elution [27]. Therefore, the UHPLC method gradient time is expressed thus:

$$ t{\text{grad}}_{2} = \frac{{(\% B{\text{final}}_{1} - \% B{\text{initial}}_{1} )}}{{slope_{2} }} $$

where *B*_*final1*_ and *B*_*initial1*_ are gradient composition of the methods involved, respectively. Hence, the gradient slope is described by:

$$ slope_{1} \cdot t_{1} = slope_{2} \cdot t_{2} $$

where t_1_ and t_2_ are the dwell times. Finally, relationship between the slopes of the methods with gradient elution of the mobile phase is as follows:

$$ slope_{2} = slope_{1} \cdot \frac{{V0_{1} }}{{V0_{2} }} \cdot \frac{{F_{2} }}{{F_{1} }} $$

## A Comparison of UHPLC and HPLC Methods

Since UHPLC and HPLC techniques exploit the same mechanism of separation, the optimization of separation conditions is based on the same principles. However, due to the application of smaller particle size fillings, high mobile phase pressure values and of velocity it is necessary to establish the equivalence of those methods. Table 1 presents the differences between assay parameters for the determination of a heterocyclic drug in organic solvent extracts by using HPLC and UHPLC methods. A method transfer from 10-min HPLC assay to 1-min UHPLC assay was performed [28].

**Table 1:** Comparison of UPLC and HPLC methods [36]

Parameters of set	HPLC method	UHPLC method
Column	XTerra C18, 50 × 4.6 mm, 4 μm particles	ACQUITY UPLC BEH C18, 50 × 2.1 mm, 1.7 μm particles
Flow rate	3.0 mL/min	0.6 mL/min
Needle wash	Methanol	Strong needle wash: 200 μL Methanol; Weak Needle Wash: 600 μL ACN:H_2_O 10:90
Injection volume	20 μL	3 μL partial loop fill or 5 μL full loop fill with automatic overfill
Gradient (time in min) (ACN: H_2_O)	T0(25:75), T6.5(25:75), T7.5(95:5), T9(25:75), T10(25:75)	T0(36:64), T1.1(95:5), T1.3(36:64)
Total run time	10 min	1.5 min
Total solvent consumption	Acetonitrile: 10.5 mL Water: 21.0 mL	Acetonitrile: 0.53 mL Water: 0.66 mL
Plate count for API	2,000	7,500
USP resolution	3.2	3.4
LOQ	~0.2 μg/mL	0.054 μg/mL
Carry-over	< 0.05 % with needle wash	0.01 %
Delay volume	~720 μL	~110 μL

The application of UHPLC and HPLC for analysis in pharmaceutical and biological matrixes has been researched in a number of studies. For example, J. Jastrebora et al. analyzed separation of folic acid derivatives by using UHPLC and HPLC methods involving BEHC18/HSST3 and X bridge C18/Atlantis C18 columns, respectively. It was found that the UHPLC method provided better sensitivity, broader linearity and satisfying separation efficiency. Analysis run-time was reduced fourfold and lower LOD values were achieved [20].

The determination of a common antineoplastic drug in the presence of 14 impurities in a pharmaceutical matrix required the use of gradient elution in both methods and identical validation parameters were obtained, with the run-time of UHPLC analysis of 34 min as compared to 90 min for HPLC [29].

Using Deming Regression analysis, Xu et al. [30] compared the determination of anticancer drug by establishing the concentration of busulfan in human plasma with UHPLC and HPLC methods. Both of them were found to meet the criteria for analytical tools used for pharmacokinetic studies (precision, reproducibility, reliability). The UHPLC method permitted a shorter time of analysis (1.3 min vs 10 min for HPLC). The derivatization and extraction of busulfan from plasma were conducted according to the same protocol.

Table 2 shows chromatograms obtained in the studies outlined above. A significant reduction of analysis run-time and of solvent consumption, greater sensitivity and a higher resolution of determination were possible to achieve by using a UHPLC method.

**Table 2:** Comparison of possibilities of determinations of selected compounds by using UPLC and HPLC methods [20, 29, 30]

	
HPLC chromatograms of busulfan (*1*) in biological matrix *Separation conditions* Stationary phase: BEH C18 (100 mm × 1.0 mm, 1.7 μm) Mobile phase: methanol:water (75:25 V/V) Flow rate: 0.14 mL/min Temp. column: ambient Detection: UV	UPLC chromatogram of busulfan (*1*) in biological matrix *Separation conditions* Stationary phase: BEH C18 (50 mm × 2.1 mm, 1.7 μm) Mobile phase: acetonitrile:water with trifluoroacetic acid (0.2 % V/V) Flow rate: 1.0 mL/min Temp. column: ambient Detection: UV
	
HPLC chromatogram of bicalutamide (*2*) and its 14 impurities in pharmaceutical dosage forms *Separation conditions* Stationary phase: Zorbax SB phenyl (150 mm × 4.6 mm, 3.5 μm) Mobile phase: acetonitrile:0.01 M dihydrogen orthophosphate:water (5:95 V/V) (gradient eluation) Flow rate: 1.0 mL/min Temp. column: 40 °C Detection: UV	UPLC chromatogram of bicalutamide (*2*) and its 14 impurities in pharmaceutical dosage forms *Separation conditions* Stationary phase: BEH C18 (100 mm × 1.0 mm, 1.7 μm) Mobile phase: methanol:water (75:25 V/V) (gradient eluation) Flow rate: 0.5 mL/min Temp. column: : 40 °C Detection: UV
	
The HPLC chromatogram of folates in dietary supplements (*3*-H_4_ folate, *4*-CH_3_-H_4_ folate, *5*-HCO-H_4_ folate) *Separation conditions* Stationary phase: XBridge C18 (150 mm × 4.6 mm, 3.5 μm)/Atlantis d18 (150 mm × 4.6 mm, 3.5 μm) Mobile phase: acetonitrile:30 mM potassium phosphate (pH = 2.3) Flow rate: 0.40 mL/min Temp. column: 23 °C Detection: UV/FL	The UPLC chromatogram of folates in dietary supplements *Separation conditions* Stationary phase: Acquity C18 (100 mm × 2.10 mm, 1.70 μm)/HSS (100 mm × 2.10 mm, 1.70 μm) Mobile phase: acetonitrile:30 mM potassium phosphate (pH = 2.3) Flow rate: 0.5 or 0.7 mL/min Temp. column: 30 °C/60 °C Detection: UV/FL

D. Guillarme et al. studied a method transfer from HPLC to UHPLC taking into account isocratic and gradient separation. The influence of the column length and the particle size of the stationary phase on the chromatographic performance was analysed. It was proven that short columns packed with sub-2 μm particles reduced the time of analysis (without affecting efficiency or resolution) more significantly than in the case of columns packed with 3.5 μm particles. The compounds involved in the study were rapidocaine and lidocaine hydrochloride in the presence of excipients. The resolution of separation with a column packed with 3.5 μm particles was not acceptable. The application of a mass spectrometry detector allowed for compatible results in simple separations conducted by using HPLC and UHPLC methods.

A method transfer from HPLC (5 μm particles) to UHPLC (a short column with 1.7 μm particles) under gradient elution conditions was also studied. A comparison of these two methods for the determination of the active substance in the presence of eleven impurities showed a significant reduction of elution time in the case of UHPLC analysis. However, efficiency, selectivity and the average retention time were worse compared to those achieved during HPLC analysis [26, 27].

## The Applicability of UHPLC

Based on a review of analytical applications of UHPLC, the technique appears to be applicable for drug determination in pharmaceutical, biological and biopharmaceutical matrixes. Examples of applying UHPLC methods in the determination of pharmaceutical substances in biological and pharmaceutical matrixes are presented in Table 3. Reports dating from 2008 to 2012, focused mainly (73 %) on the application of UHPLC methods for the analysis of pharmaceutical substances in their dosage forms.

**Table 3:** Possibilities of application of UPLC and HPLC in analysis of active pharmaceutical ingredients in pharmaceutical and biological matrices

The aim of studies/material	Conditions of separations	References
*Pharmaceutical matrix*		
Analysis of active pharmaceutical ingredients in bulk substances		
*Simultaneously determination of drugs* Determination of β-blokers: Atenolol, pindolol, acebutolol, metoprolol, oxprenolol, propranolol, alprenolol	Stationary phase: BEH C18 (100 mm × 2.1 mm, 1.7 μm) Mobile phase: A solvent: 0.1 % trifluoroacetic acid in water; B solvent: 0.1 % trifluoroacetic acid in acetonitrile Flow rate: 0.5 mL/min Temp. column: ambient Detection: MS Injection volume: 5 μL	[32]
*The determination of drug in the presence of its impurities* Determination of primaquine phosphate in presence of two impurities	Stationary phase: BEH C18 (50 mm × 2.1 mm, 1.7 μm) Mobile phase: 0.01 % trifluoroacetic acid: acetonitrile (75:25 V/V) Flow rate: 1 mL/min Temp. column: 35^° ^C Detection: UV Injection volume: 10 μL	[33]
Determination of esomeprazole magnesium trihydrate in the presence of seven impurities	Stationary phase: BEH C18 (50 mm × 2.1 mm, 1.7 μm) Mobile phase: A solvent: 0.04 M glycine (pH = 9.0) buffer; B solvent: acetonitrile (90:10 V/V) Flow rate: 0.21 mL/min Temp. column: ambient Detection: UV Injection volume: 2.8 μL	[34]
*The determination of drug in the presence of its degradation products* Determination of febuxostat in presence of degradation products	Stationary phase: BEH C18 (150 mm × 2.1 mm, 1.7 μm) Mobile phase: ammonium acetate buffer (pH = 4.5): acetonitrile (70:30 V/V) Flow rate: 0.2 mL/min Temp. column: 30 °C Detection: UV Injection volume: 2 μL	[35]
*The determination of drugs in the presence of their degradation products* Determination of losartan potassium, atenolol, hydrochlothiazide in presence of degradation products	Stationary phase: Zorbax C18 (50 mm × 4.6 mm, 1.8 μm) Mobile phase: water: triethylamine: orthophosphoric acid: acetonitrile (60:0.1:0.1:30 V/V/V/V) Flow rate: 0.7 mL/min Temp. column: 25 °C Detection: UV Injection volume: 5 μL	[36]
*The determination of drug in the presence of their degradation products and impurities* Determination of ranolazine in presence of degradation products and impurities	Stationary phase: BEH C18 (100 mm × 2.1 mm, 1.7 μm) Mobile phase: A solvent: 0.01 M acetonitrile:triethylamine (pH = 7.3): sodium dihydrogen phosphate (10:90:0.1 V/V/V); B solvent: acetonitrile (55:45 V/V) Flow rate: 0.3 mL/min Temp. column: 27 °C Detection: UV Injection volume: 1 μL	[37]
Analysis of active pharmaceutical ingredients in pharmaceutical dosage forms		
*The determination of drug in the presence of its degradation products* Determination of imatinib mesylate	Stationary phase: BEH C18 (50 mm × 2.1 mm, 1.7 μm) Mobile phase: A solvent: 0.05 M ammonium acetate (pH = 9.5); B solvent: acetonitrile and methanol (40:60 V/V) Flow rate: 0.3 mL/min Temp. column: 30 °C Detection: UV Injection volume: 2.0 μL	[38]
*The determination of drugs in the presence of their degradation products* Determination of atorvastatin, fenofibrate and their degradation products	Stationary phase: BEH C18 (100 mm × 2.1 mm, 1.7 μm) Mobile phase: A solvent: 0 (pH = 4.7); B solvent: acetonitrile Flow rate: 0.5 mL/min Temp. column: ambient Detection: UV Injection volume: 1.0 μL	[39]
Analysis of dietary supplements ingredients		
*The determination of dietary supplements* Determination of 12 hoodigosides in *Hoodia sp.*	Stationary phase: BEH C18 (100 mm × 2.1 mm, 1.7 μm) Mobile phase: A 0.05 % formic acid; B solvent: acetonitrile Flow rate: 0.35 mL/min Temp. column: 20 °C and 40 °C Detection: UV Injection volume: 5.0 μL	[40]
Determination of 11 saponins in *Panax notoginseng*	Stationary phase: BEH C18 (50 mm × 2.1 mm, 1.7 μm) Mobile phase: A water; B solvent: acetonitrile Flow rate: 0.35 mL/min Temp. column: 45 °C Detection: UV Injection volume: 1.0 μL	[41]
Determination of 5 folates in dietary supplements	Stationary phase: BEH C18 (50 mm × 2.1 mm, 1.7 μm) Mobile phase: A water; B solvent: acetonitrile Flow rate: 0.35 mL/min Temp. column: 45 °C Detection: UV Injection volume: 1.0 μL	[29]
*Biological matrix*		
Analysis of active pharmaceutical ingredients in biological fluids		
*The determination of substance obtained by biotechnological synthesis* Determination of cefuroxime lysine in dog plasma	Stationary phase: BEH C18 (50 × 2.1 mm, 1.7 μm) Mobile phase: acetonitrile: 0.1 % formic acid in 10 mM ammonium acetate (40:60 V/V) Flow rate: 0.2 mL/min	[42]
*The determination of substance obtained by chemical synthesis* Determination of busulfan in human plasma	Temp. column: 25 °C Detection: MS/MS Injection volume: 10.0 μL Stationary phase: BEH C18 (50 mm × 2.1 mm, 1.7 μm) Mobile phase: acetonitrile: 0.2 % trifluoroacetic acid in water Flow rate: 1.0 mL/min Temp. column: ambient Detection: UV Injection volume: 5.0 μL	[30]
*The determination of endogenic substance* Determination of erythropoietin in human serum albumin	Stationary phase: C18 (50 mm × 2.1 mm, 1.7 μm) Mobile phase: 0.1 % TFA in acetonitrile: 0.1 % TFA in water (gradient elution) Flow rate: 0.35 mL/min Temp. column: 60 °C Detection: UV Injection volume: 2.0 μL	[43]

Due to a good resolution and sensitivity it was possible to use UHPLC method in the determination of analytes in bulk substances as well as in pharmaceutical dosage forms. Applications of UHPLC methods concerned assays of compounds with similar chemical structures, for example analogues from the same therapeutic group. Such methods can also be applied in the presence of related substances, including impurities and degradation products (Table 3) [31–43]. The most common applications of UHPLC methods in pharmaceutical analysis in recent years have dealt with:

- Stability studies of API in bulk substance as well as in its pharmaceutical forms, especially during the development of stability-indicating analytical methods [37–39, 44–48]
- Profiles of impurities [49, 50]
- Dissolution studies [41, 51, 52].

As those areas of research require a large amount of API determination, a widespread use of UHPLC methods may help to solve the problem of excessive time and solvent consumption while maintaining adequate resolution and sensivity. The UHPLC methods can be used in analysis of API in the biological matrixes involving identification of metabolites and bioequivalence studies in biological fluids [53–58]. Ultimately, the determination of API at the lowest possible concentration levels may be achieved by the coupling of UHPLC with the mass spectrum detector [59].

## Conclusions

In light of the benefits discussed in this review, the application of UHPLC in pharmaceutical analysis may be considered a greening pathways for liquid chromatography, which is especially significant for drug analysis in the pharmaceutical matrix. Also UHPLC may be applied in stability studies, when the required number of determinations is especially high, with the advantage of reducing the amount of organic solvents and the concentration of analytes.

Ultra-high-performance liquid chromatography appears to have the potential to replace the less environment-friendly analytical techniques provided that methods based on this kind of chromatography have been properly validated. Modifications of UHPLC methods will probably aim at the elimination of friction heating by looking for new solutions in the development of stationary and mobile phases.
